# The Needs for Visual Improvement of Patients Presented at Low-Vision Center in Wenzhou, China

**DOI:** 10.1155/2019/3586370

**Published:** 2019-08-28

**Authors:** Xiaoman Li, Guofu Chen, Ruzhi Deng, Na Lin, Lingzhi Ni, Longfei Jiang, Haishuang Lin, Frank Thorn, Jie Chen

**Affiliations:** ^1^School of Optometry and Ophthalmology and Eye Hospital of Wenzhou Medical University, Wenzhou, Zhejiang, China; ^2^Zhejiang Eye Hospital at Zhijiang, Hangzhou, Zhejiang, China; ^3^Department of Brain and Cognitive Sciences, Massachusetts Institute of Technology, 77 Massachusetts Avenue, Cambridge, MA 02139, USA

## Abstract

**Purpose:**

To characterize the needs for visual improvement of new-visit patients with low vision.

**Methods:**

This cross-sectional study collected detailed information of patients presented at low-vision center of the Eye Hospital of Wenzhou Medical University between January 2015 and January 2017. A questionnaire interview, including demographic information and needs for visual improvement, was conducted before ophthalmology examinations.

**Results:**

The main need for visual improvement was engagement in hobbies (68.9%), followed by reading (20.9%), engaging in occupation (20.1%), and watching TV or movies (17.1%). Less than 10% of patients mentioned the demand of using public transportation (5.8%), doing housework (3.7%), writing (1.9%), walking on irregular surfaces (1.5%), driving (1.1%), and others (2.4%). Women were significantly associated with a concern for performing hobbies (OR 1.45, 95% CI 1.0–2.0) but associated with lower odds of reading (OR 0.46, 95% CI 0.3–0.7). Older subjects were more willing to choose hobbies (OR 1.35 (per 10-year increase), 95% CI 1.3–1.4), reading (OR 1.11 (per 10-year increase), 95% CI 1.0–1.2), watching TV or movies (OR 1.4 (per 10-year increase), 95% CI 1.3–1.6), and housework (OR 1.21 (per 10-year increase), 95% CI 1.0–1.5) than younger individuals. In comparison with younger participants, older individuals were less likely to choose occupation (OR 0.53 (per 10-year increase), 95% CI 0.5–0.6). No significant association was found between visual acuity and needs for visual improvement.

**Conclusion:**

Hobbies, reading, engaging in occupation, and watching TV were the most common needs for visual rehabilitation in patients with visual impairment. Gender and age showed a modest influence on the choice of different needs.

## 1. Introduction

Visual impairment caused by a variety of ocular diseases has a strong impact on function, physics, and psychology of people. It limits the performance of daily living activities [1], decreases quality of life [2, 3], and affects mood [4–8]. According to the 2010 World Health Organization report, there were estimated 75 million people who were visually impaired in China. At present, low-vision rehabilitation is essential for patients with visual impairment. It enables patients to perform daily activities and improve social function through the use of low vision aids and learning to manage daily activities [9–11]. The ultimate aim of low-vision rehabilitation is to help patients to live an independent life like people with normal vision.

Low-vision rehabilitation is individually designed depending on patients' needs and complaints which vary tremendously. In America, reading, driving, watching movies, sports, and work-related duties were the most important needs of patients with visual impairment [12–16]. However, there is little research addressing the needs for visual improvement of visually impaired patients in China [17]. For a better understanding of the needs of Chinese patients with visual impairment, we did this study.

## 2. Materials and Methods

This is a cross-sectional study. All patients who present to our low-vision center would undergo comprehensive visual function evaluations before the rehabilitation plan was given. Both subjective and objective evaluations were involved. Because rehabilitation was a very personal process, so we emphasized the subjective evaluations in our low-vision center. The subjective evaluation was a 30–40 min face-to-face interview conducted by two nurses (LFJ and LZN). It included demographic information, medical history, rehabilitation needs for visual improvement, and questionnaires. Demographic information includes age, gender, occupation, address, and contacts. Visual needs were the visual functions that patients really wanted to improve. Several questionnaires were used in the clinic according to different patients such as Elderly Low Vision Quality of Life Questionnaire (ELVQoL) [17], Low Vision Quality of Life Questionnaire, and Visual Function-25, of which ELVQoL was the most commonly used. Children less than 10 years old were accompanied by guardians and they answered questions together. Objective evaluations consisted of habitual visual acuity (VA) tests and best-corrected visual acuity (BCVA) (Early Treatment Diabetic Retinopathy Study or Feinbloom) tests, refraction, contrast sensitivity tests, visual field tests, slit-lamp examination, and fundus examinations, which were all performed by low-vision specialists in the center. At last, the diagnoses would be made, and the visual impairment level was defined according to the categories of the International Classification of Diseases 2008 version [18]. The ocular disease considered to be primarily responsible for the visual loss was identified for patients with visual impairment. Finally, an individual rehabilitation plan would be recommended to patients. In this study, patients whose BCVA is less than 6/12 were included. The study adhered to the tenets of the Declaration of Helsinki. Verbal informed consent was obtained from study participants or legal guardians.

Descriptive statistics were performed to compare needs for visual improvement of the gender groups, age groups, and BCVA groups. Data are presented as mean and standard deviation. Chi-square analyses were used to compare item results across genders, age, and BCVA, respectively. Multivariate logistic regression models were performed to assess the relevance of age, gender, and BCVA with regard to the likelihood of needs for visual improvement in each item. Statistical significance was defined as *P* < 0.05. Statistical analyses were performed using the SPSS 22.0 (SPSS Inc., Chicago, Illinois, USA).

## 3. Results

Records of 736 first-visit patients between January 2015 and January 2017 were received, 391 (53.1%) were males, and 345 (46.9%) were females. The age range was from 2 to 95 years (median age was 50 years). The majority of patients (347, 47.1%) had moderate visual impairment (6/18>–≥6/60). The percentage of patients with mild visual impairment (≥6/18) and severe visual impairment (6/60>–≥3/60) was 17.1% (126) and 16.0% (118), respectively. In this context, the percentage of low-vision patients with moderate or severe visual impairment was 63.2% (465/736), and the percentage of the blind (<3/60) was 19.7% (145/736).

**Figure 1:**
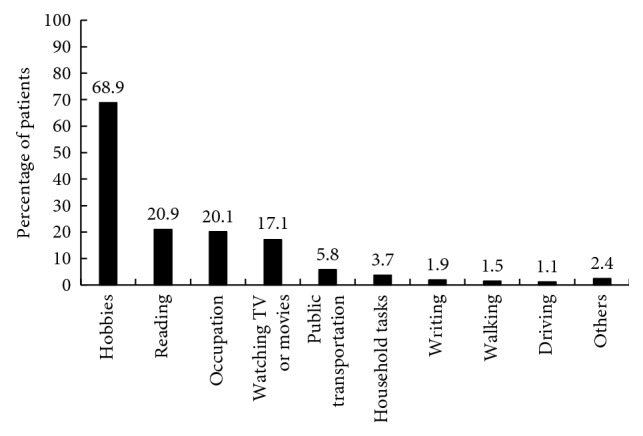
Frequency of needs for visual improvement. Multiple choices were permitted, and therefore, the total category may exceed 100%.

The main needs for patients with visual impairment was engagement in hobbies (507, 68.9%) such as playing chess, playing cards, playing mahjong, and building blocks, followed by reading (154, 20.9%), engaging in occupation (148, 20.1%), and watching TV or movies (126, 17.1%) (Figure 1).

Males were more likely to choose reading (26.1% vs. 15.1%; *P* < 0.001) and occupation (24.0% vs. 15.7%; *P*=0.005) as their primary need for visual rehabilitation, compared to females, while females were more likely to choose performing hobbies (74.5% vs. 63.9%; *P*=0.002) and doing housework (5.2% vs. 2.3%; *P*=0.036) (Table 1).

Patients were divided into three groups according to adolescents under the age of 18 (1- to 17-year age band), labor force (18- to 64-year age band), and old people (≥65-year age band). Patients aged 65 (*n* = 195) or over were more likely to choose engaging in hobbies (81.0% vs. 71.6%, *P* < 0.017; 81.0% vs. 43.9%, *P* < 0.017) and watching TV/movies (27.2% vs. 17.7%, *P* < 0.017; 27.2% vs. 1.4%, *P* < 0.017) than one who were 18–64 (*n* = 402) and 1–17 years old (*n* = 139). 1- to 17-year-old age band were more inclined to select occupation (55.4% vs. 16.9%, *P* < 0.017; 55.4% vs. 1.5%, *P* < 0.017) than participants who were 18–64 and ≥65 years old. Besides, the median age of people who chose engaging in occupation (17 years old) was significantly less than those who chose hobbies (55 years old), reading (51 years old), watching TV/movies (62 years old), public transportation (49 years old), household tasks (59 years old), walking on irregular surfaces (stairs, steps, and curbs) (62 years old), and others (51 years old) (*P* < 0.05). The proportion of reading in 1- to 17-year age band was less than 18-to 64- and ≥65-year age band (11.5% vs. 22.6%, *P* < 0.017; 11.5% vs. 24.1%, *P* < 0.017). The proportion of using public transportation in 1- to 17-year age band was less than 18- to 64-year age band (0.7% vs. 8.0%, *P* < 0.017). Other needs were not significantly different between different age bands (Table 2).

The blind chose engagement in hobbies more than those with mild visual impairment (80.0% vs. 65.9%, *P* < 0.017) and the low-vision (moderate and severe visual impairment) patients (80.0% vs. 66.2%, *P* < 0.017). Patients with mild visual impairment were more likely to choose reading than the low-vision patients (29.4% vs.19.4%, *P* < 0.017). Additionally, the low-vision patients were inclined to choose engaging in occupation more than the mildly visually impaired patients (25.4% vs. 14.3%, *P* < 0.017) and the blind (25.4% vs. 8.3%, *P* < 0.017) (Table 3).

Results of the multiple logistic regression analyses for the association of age, gender, and BCVA with each type of needs are shown in Table 4. Women were significantly associated with a concern for performing hobbies (OR: 1.45, 95% CI: 1.0–2.0) but were associated with lower odds for reading (OR: 0.46, 95% CI: 0.3–0.7). Older subjects were more willing to choose engaging in hobbies (OR: 1.35, 95% CI: 1.3–1.4 for 10-year increase in age), reading (OR: 1.11, 95% CI: 1.0–1.2 for 10-year increase in age), watching TV or movies (OR: 1.4, 95% CI: 1.3–1.6 for 10-year increase in age), and housework (OR: 1.21, 95% CI: 1.0–1.5 for 10-year increase in age) than younger people. In comparison with younger participants, older people were less likely to choose occupation (OR: 0.53, 95% CI: 0.5–0.6 for 10-year increase in age). No significant association was found between BCVA and needs for visual improvement (Table 4).

## 4. Discussion

### 4.1. Hobbies

In this study, the need for engaging in hobbies (68.9%) was overwhelmingly greater than other needs for visual improvement. The need for reading, occupation, and others were all less than 30%. In patients with different diseases, engagement in hobbies was also the most common need, except for nystagmus patients. This result indicates that hobbies are very important for the visually impaired populations. Previous studies showed that participations in leisure activities had a positive correlation with the quality of life, and hobbies were the strongest predictor [19]. Some studies have also indicated that leisure activities can improve individual well-being [20] and self-esteem [21]. These results may explain why so many patients chose engagement in hobbies as their need for visual rehabilitation.

Previous studies concluded that women generally took more responsibility in looking after children and doing housework. As a result, females spent less free time in leisure activities than males [22]. In our study, females were more likely to choose hobbies as a need for visual improvement than males. A great deal of housework may make women want to participate in leisure activities more so as to relieve the pressure of life [23].

The probability of choosing hobbies as the main need for visual rehabilitation slightly increased with older age. In the study, nearly half of the participants had reached the retirement age. Marginalization and the decline in social status caused a decline in the quality of life. Less satisfying personal lives made the elder eager to participate in hobbies. Besides, more leisure time was available to the retired elder which gave them more opportunities to participate in hobbies.

### 4.2. Reading

Goldstein and colleagues found that reading was the most frequent complaint and was reported by 66.4% American low-vision patients in clinics. In contrast, only 20.9% of outpatients in this study wanted to improve their reading ability. This may be related to difference in reading habits between two countries. In 2016, an American read an average of 12 books per year [24]. However, the number was only 7.84 in China. The gap in reading needs may also occur in low-vision patients in both countries.

In China, the reported illiteracy rate for females and males aged 15 and above was 8.01% and 2.89%, respectively, which showed that the imbalance in education caused by gender bias still existed [25]. Another study also found that men in China had more educational opportunities than women [26]. The problem of imbalanced education due to gender differences may also be reflected in outpatient attitudes toward reading.

The findings showed that older individuals had a greater likelihood of choosing reading as their need for visual rehabilitation. This result is consistent with previous studies [12, 16]. Additionally, the possibility of choosing housework also increased slightly with age. However, the small magnitude in both findings suggests they have limited clinical significance.

### 4.3. Occupation

Visually handicapped people have been hindered in their occupation development. Ganesh et al. found that the most important difficulty for visually impaired students was related to academic activities like copying from the blackboard and reading textbook at arm's length [14]. These difficulties undoubtedly affect the patient's knowledge acquisition. Furthermore, visual handicaps also created obstacles to career development, such as lower education and a lack of working experiences and skills [27, 28]. The impact of visual impairment was undoubtedly more pronounced for the elderly with a diminished ability to work. And on the other hand, the decreased working ability affected the attitudes of the public toward the employment of visually impaired persons.

In addition, the traditional idea that men's work centers always around the outside, whereas women's work centers always around the home leads to the phenomenon that there is a greater proportion of male workers than female workers [29]. The culture also had an influence on job-seeking behavior of low-vision patients. In these findings, men were more likely to choose engaging in occupation than women. Conversely, females preferred choosing engagement in doing housework. But there was no statistical difference between females and males in the logistic regression.

### 4.4. Driving

Compared to the western countries, fewer patients in this study population had a demand for driving. Different cultures and economic levels may explain this difference. Rich car culture and strong purchasing power made the American per-capita car ownership much higher than the Chinese. According to the 2016 report of Organization International des Constructeurs d'Automobiles, the United States has 821 cars per 1,000 people, compared to 118 in China [30]. In addition, it is also related to the different standards of the driver's license examinations carried out by the two countries. In the United States, most standards are that the BCVA of a driver must be 20/40 in both eyes or in the better eye, with/without corrective lenses. The state governments also specifically set appropriate driving standards in order to improve the independence of patients with low vision [31]. In comparison with America, there is a more stringent standard for driving in China [32] (uncorrected VA or corrected VA must be better than 20/25). Furthermore, there is no driving standard specifically designed for low-vision patients. Ultimately, both facts reduced low-vision patient's interest in driving.

### 4.5. Limitation

There are a number of limitations in this study. The study used the convenience sampling which cannot represent all population with visual impairment. The questionnaire interview is based on the ELVQoL [17], so does not include the specific needs such as education for children. Despite these limitations, this study is among the first to investigate the needs for visual improvement of patients with visual impairment in China.

## 5. Conclusions

Hobbies, reading, engaging in occupation, and watching TV were the most common needs for visual rehabilitation in patients with visual impairment. BCVA was not associated with visual rehabilitation needs, but gender and age showed a modest influence on the choice of different needs for visual impairment.

## Figures and Tables

**Table 1:** Needs for visual improvement for low-vision patients between genders.

Type of needs	Men (%)	Women (%)	*P* value
Hobbies	**63.9**	**74.5**	**0.002**
Reading	**26.1**	**15.1**	<**0.001**
Occupation	**24.0**	**15.7**	**0.005**
Watching TV or movies	16.4	18.0	0.565
Public transportation	5.4	6.4	0.561
Household tasks	**2.3**	**5.2**	**0.036**
Writing	2.8	0.9	0.054
Walking on irregular surfaces (stairs, steps, and curbs)	1.3	1.7	0.607
Driving	1.8	0.3	0.109
Others (such as makeup, shopping, and recognizing faces)	2.6	2.3	0.834

*P* value less than 0.05 was listed in bold.

**Table 2:** Needs for visual improvement for low-vision patients among different age bands.

Type of needs	1–17 (%)	18–64 (%)	≥65 (%)	*P* value
Hobbies	**43.9**	**71.6**	**81.0**	**<0.001**
Reading	**11.5**	**22.6**	**24.1**	**0.009**
Occupation	**55.4**	**16.9**	**1.5**	**<0.001**
Watching TV or movies	**1.4**	**17.7**	**27.2**	**<0.001**
Public transportation	**0.7**	**8.0**	**5.1**	**0.006**
Household tasks	0.7	3.7	5.6	0.063
Writing	2.9	2.0	1.0	0.407
Walking on irregular surfaces (stairs, steps, and curbs)	0	2.2	1.0	0.179
Driving	0.0	1.7	0.5	0.232
Others (such as makeup, shopping, and recognizing faces)	1.4	3.2	4.8	0.418

*P* value less than 0.05 was listed in bold.

**Table 3:** Needs for visual improvement for low-vision patients by visual acuity.

Type of needs	Mild visual impairment: ≥6/18 (%)	Low-vision: 6/18>–≥3/60 (%)	Blind: 3/60>–≥NLP (%)	*P* value
Hobbies	**65.9**	**66.2**	**80.0**	**0.005**
Reading	**29.4**	**19.4**	**18.6**	**0.038**
Occupation	**14.3**	**25.4**	**8.3**	<**0.001**
Watching TV or movies	23.0	16.3	14.5	0.136
Public transportation	7.1	5.4	6.2	0.739
Household tasks	4.0	3.2	4.8	0.657
Writing	**4.0**	**1.9**	**0.0**	**0.035**
Walking on irregular surfaces (stairs, steps, and curbs)	1.6	1.3	2.1	0.681
Driving	0.0	1.5	0.7	0.521
Others (such as makeup, shopping, and recognizing faces)	2.4	2.2	3.4	0.578

*P* value less than 0.05 was listed in bold.

**Table 4:** Associations in needs for visual improvement after adjusting for gender, age, and visual acuity.

Type of needs	Independent variables: odds ratio (95% CI)		
	Female	Age (per 10 yrs)	BCVA (0.1 logMAR worse)
Hobbies	**1.45** (**1**.**0**–**2**.**0**)	**1.35** (**1**.**3**–**1**.**4**)	1.06 (0.7–1.5)
Reading	**0.46** (**0**.**3**–**0**.**7**)	**1.11** (**1**.**0**–**1**.**2**)	0.96 (0.7–1.4)
Occupation	0.72 (0.5–1.1)	**0.53** (**0**.**5**–**0**.**6**)	0.93 (0.6–1.6)
Watching TV or movies	0.92 (0.6–1.4)	**1.40** (**1**.**3**–**1**.**6**)	0.95 (0.6–1.4)
Public transportation	1.18 (0.6–2.2)	1.03 (0.9–1.2)	1.01 (0.6–1.8)
Household tasks	2.09 (0.9–4.8)	**1.21** (**1**.**0**–**1**.**5**)	1.05 (0.6–1.9)
Writing	0.31 (0.1–1.1)	0.90 (0.7–1.1)	0.76 (0.1–6.7)
Walking on irregular surfaces (stairs, steps, and curbs)	1.20 (0.4–1.0)	1.20 (0.9–1.7)	1.02 (0.4–2.8)
Driving	0.16 (0.02–1.3)	0.99 (0.7–1.3)	0.96 (0.2–4.5)
Others (such as makeup, shopping, and recognizing faces)	0.88 (0.3–2.3)	1.10 (0.9–1.3)	1.05 (0.5–2.2)

CI = confidence interval; BCVA = best-corrected visual acuity; logMAR = logarithm of the minimum angle of resolution. *P* value less than 0.05 was listed in bold.

## Data Availability

The data used to support the findings of this study are included within the article.
